# Distance-based paper device using combined SYBR safe and gold nanoparticle probe LAMP assay to detect *Leishmania* among patients with HIV

**DOI:** 10.1038/s41598-022-18765-w

**Published:** 2022-08-26

**Authors:** Toon Ruang-areerate, Natkrittaya Saengsawang, Panthita Ruang-areerate, Nalin Ratnarathorn, Thanyapit Thita, Saovanee Leelayoova, Suradej Siripattanapipong, Kiattawee Choowongkomon, Wijitar Dungchai

**Affiliations:** 1 Department of Parasitology, Phramongkutklao College of Medicine, Bangkok, 10400 Thailand; 2 Analytical Chemistry, Department of Chemistry, Faculty of Science, King Mongkut’s University of Technology Thonburi, Bangkok, 10140 Thailand; 3 National Omics Center, National Science and Technology Development Agency (NSTDA), Pathum Thani, 12120 Thailand; 4 Research Division, Office of Police Strategy, Royal Thai Police, Bangkok, 10330 Thailand; 5 Department of Microbiology, Faculty of Science, Mahidol University, Bangkok, 10400 Thailand; 6 Department of Biochemistry, Faculty of Science, Kasetsart University, Bangkok, 10900 Thailand

**Keywords:** Microbiology, Molecular biology, Diseases, Medical research, Molecular medicine

## Abstract

Asymptomatic visceral leishmaniasis cases increase continuously, particularly among patients with HIV who are at risk to develop further symptoms of leishmaniasis. A simple, sensitive and reliable diagnosis is crucially needed due to risk populations mostly residing in rural communities with limited resources of laboratory equipment. In this study, a highly sensitive and selective determination of *Leishmania* among asymptomatic patients with *Leishmania/*HIV co-infection was achieved to simultaneously interpret and semi-quantify using colorimetric precipitates (gold-nanoparticle probe; AuNP-probe) and fluorescence (SYBR safe dye and distance-based paper device; dPAD) in one-step loop-mediated isothermal amplification (LAMP) assay. The sensitivities and specificities of 3 detection methods were equivalent and had reliable performances achieving as high as 95.5%. Detection limits were 10^2^ parasites/mL (0.0147 ng/µL) which were 10 times more sensitive than other related studies. To empower leishmaniasis surveillance as well as prevention and control, this dPAD combined with SYBR safe and gold nanoparticle probe LAMP assay is reliably fast, simple, inexpensive and practical for field diagnostics to point-of-care settings in resource-limited areas which can be set up in all levels of healthcare facilities, especially in low to middle income countries.

## Introduction

Leishmaniasis is a vector-borne disease caused by flagellate protozoan parasites of the genus *Leishmania* remaining one of the global health challenges affecting millions of people worldwide. The parasite is transmitted by the bite of female blood feeding phlebotomine sandfly. More than 1 billion people live in leishmaniasis endemic areas where approximately 0.9 and 1.7 million people are newly infected. Only a small fraction will further develop the disease and 20,000 to 30,000 will eventually die^1^. The manifestations range from self-healing cutaneous leishmaniasis (CL) to potentially fatal outcome, visceral leishmaniasis (VL). In Thailand, the number of cases has continuously increased among patients with HIV/AIDS living in northern and southern regions due to the emergence of CL and VL caused by *L. (Mundinia) martiniquensis* and *L. (Mundinia) orientalis*, originally reported as *L. (Mundinia) siamensis*^2–4^. Remarkably, HIV probably influences the transmission of leishmaniasis in the affected areas due to a decline in host immune response.

The primary diagnostic test for HIV co-infection cases is currently polymerase chain reaction (PCR) because the method is trustworthy with high sensitivity and specificity^3,5^. However, PCR may be less practical and inappropriate in field diagnostics to point-of-care settings because it requires sophisticated and well-maintained laboratory equipment. Additionally, due to prolonged turnaround times of PCR operating procedures, the detection step may require 5 to 8 h or more^6^. On the contrary in terms of simplicity, the loop-mediated isothermal amplification (LAMP) assay is a simple approach to rapidly amplify large amounts of DNA within an hour by strand displacement activity of *Bst* polymerase based on isothermal conditions setting between 60 and 65 °C, and using a set of 4 to 6 primers without the use of complicated equipments^7–9^.

Several detection methods including turbidity, fluorescence and color have been developed to simply visualize and measure LAMP products using the naked eye^10–16^. To avoid the common contamination prone during the postamplification preparation step in the LAMP assay, a fluorescent detection reagent (FDR) is occasionally used in a prereaction^17–19^. A low cost and nonmutagenic fluorescent dye, SYBR safe, was used recently to reduce ambiguous evaluation in prereaction preparation; however, a fluorescent light source was needed to visualize the LAMP amplicons^14^.

Alternately, to avoid ambiguous reading and mutagenic effect, the LAMP products can be traced with functionalized gold nanoparticles (AuNPs) and analyzed using colorimetric detection^15,20–23^. Based on bio-nanoprobes, Thiol-linking of DNA and chemical functionalization of AuNPs for specific protein/antibody binding, simple and inexpensive methods were commonly applied to detect specific DNA sequences and are presently being expanded to diverse fields of pathogen diagnosis^24–28^. After hybridization with complementary DNA targets, the red solution indicating positive results remains because the complementary targets and AuNP probes form the polymeric network of cross-linked AuNPs preventing them from aggregation by salt induction. On the other hand, non-crosslinking hybridization induces aggregation of the AuNP probes by increasing salt concentration and results in a color change from red to blue (negative result)^29,30^ for which unambiguous interpretation of LAMP targets can be clearly observed by the naked eye^13,15^.

In 2017, a novel distance-based paper device (dPAD) using LAMP method was initially developed for detecting *E. coli*^31^. Less Mg^2+^ indirectly correlated to the distance on the paper device demonstrating a high concentration of LAMP products. Unreliable interpretation was observed when Mg^2+^ concentration was initially over or below narrow ranges of Mg^2+^ detection of the device. The dPAD could detect DNA lower than 10^3^ copies/µL using several preparation steps; at least, and a 25 µL loading sample was required. According to indirect detection, visualization using direct DNA binding dyes, for example, SYBR Green, EvaGreen and SYBR safe indicators^10,14,32,33^, could be more reliable and preferable as an appropriate choice for dPAD. Recently, fluorescent dPAD using LAMP method has been reported to semi-quantify *E. coli* in urine and showed considerable promise for use as a fast and inexpensive UTI (urinary tract infection) diagnostics option due to fewer procedures to prepare using simple equipment^12^. Indeed, the approach could detect *E. coli* in less than 2 h without the postreaction staining process which was faster than conventional culture as well as the PCR method. Moreover, the length of fluorescent migratory distance directly correlated with the amount of LAMP products and reflected initial *E. coli* concentration. In addition to *Bst* polymerase, recent innovative works that combined the use of AuNPs with Recombinase Polymerase Amplification (RPA) isothermal amplification could also be used for *Leishmania* detection in animals using electrochemical and paper-based detection^34,35^.

In this present study, we reported a broad application of LAMP technique in a one-step assay that could be used to screen the infection as well as semi-quantify the *Leishmania* parasites in the buffy coat collected from patients with *Leishmania*/HIV co-infection. The development of a simplified and inexpensive dPAD combined with SYBR safe and gold‑nanoparticle probe LAMP assay was demonstrated that allowed rapid interpretation within a few minutes with high sensitivity and specificity.

## Results

### Semi-quantitative SYBR safe and AuNP-LAMP assay using dPAD

**Figure 1:**
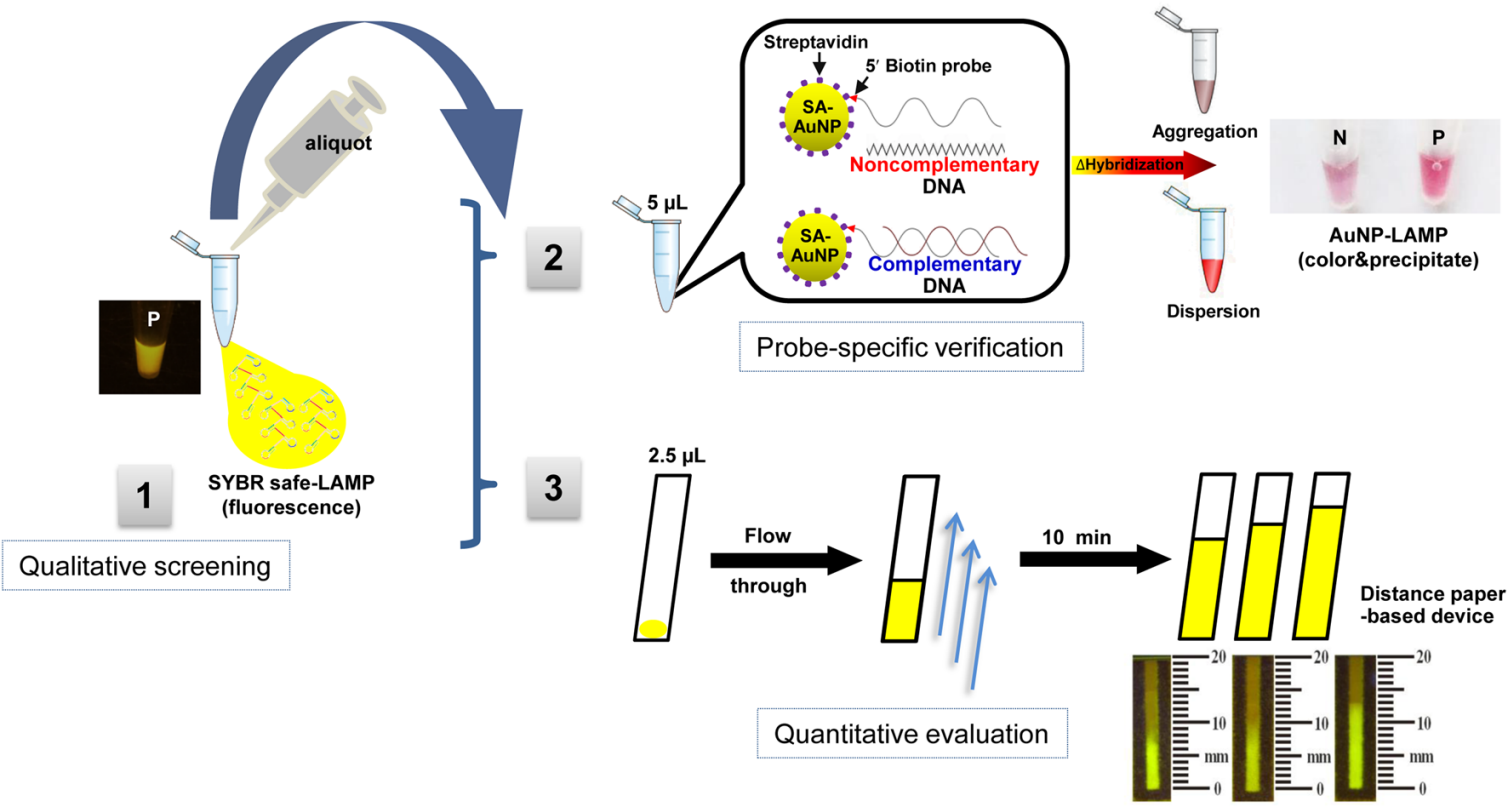
Schematic procedure of LAMP detection and semi-quantification using a distance-based paper device combined with one-step SYBR safe and gold‑nanoparticle probe LAMP assay.

A schematic of the experimental principle and signal detection to visualize LAMP products in prereaction detection using fluorescent SYBR safe for rapid screening, and to visualize and quantify LAMP products in postamplification detection using LAMP probe-streptavidin conjugated AuNPs (SA-AuNP) for specific colorimetric precipitate induction as well as a distance-based paper device (dPAD) for semi-quantifying LAMP amplicons using DNA immobilized cellulose Whatman No.1, respectively, are shown in Fig. 1. Rapid qualitative screening could be done by closed tube SYBR safe LAMP method and directly visualized by the naked eye without a requirement of postamplification preparation step using blue light (BL) transilluminator (1). To re-evaluate and verify the first screening detection, 5 µL of LAMP reaction was transferred to a new tube for colorimetric precipitate detection by post hybridization of an AuNP-probe (2). Three processes were included, that is, complementary hybridization of the single strand probe-SA-AuNP to LAMP target, colorimetric and precipitate induction by MgSO_4_ salt and detection of colorimetric precipitate by the naked eye. The polymeric network of cross-linked AuNPs is formed according to the hybridization of the single strand probe-SA-AuNP that prevents the aggregation by salt induction, leading to unchanged red color of the reaction mixture. Conversely, the reaction mixture turns to pale purple with solid precipitates due to an absence of the LAMP targets that leads the aggregation of free probe-SA-AuNPs. The dPAD can be performed simultaneously to evaluate the degree of *Leishmania* infection in the samples by pipetting 2.5 µL of LAMP reaction of the closed tube SYBR safe LAMP method to the sample zone of the dPAD and waiting for 10 min (3). This simple process of detection includes LAMP reaction flowing into the channel of the detection zone, anionic DNA immobilized by cellulose Whatman No.1 filter paper, and distance measurement of the immobilized LAMP amplicons by the naked eye under BL transilluminator. The fluorescence of migratory distance correlates to the final amounts of LAMP products. DNA is a strongly anionic molecule and could be immobilized on the cellulose paper due to the physical adsorption and the charge interaction between DNA and the immobilized cationic polymers.

### Optimization of dPAD for quantitative LAMP assay

**Figure 2:**
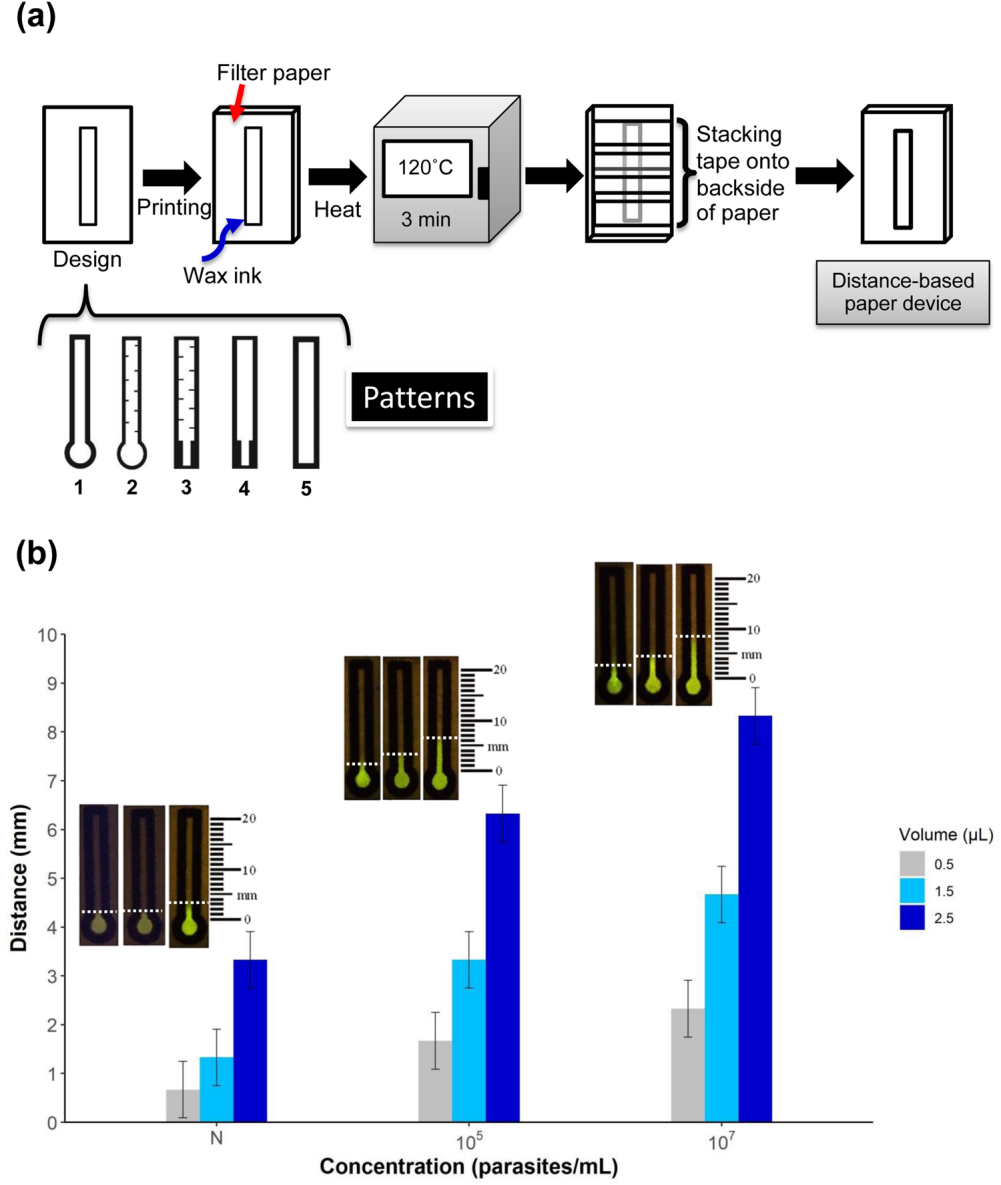
Fabrication process of a distance-based paper device (dPAD) and optimization of load sample volume, (**a**) schematic illustration of fabrication process, (**b**) fluorescent migratory distance of SYBR safe on the dPAD at different load volumes of LAMP reaction mixture (0.5, 1.5 and 2.5 µL, respectively): negative (N); no *Leishmania* DNA control, positives (10^5^ and 10^7^); 10^5^ and 10^7^ parasites/mL of *Leishmania* DNA.

The illustration of fabrication process is shown in Fig. 2a. After inactivating penetration of the hydrophobic wax ink and installing transparent adhesive tape to limit the boundary channel of sample and detection zones of the dPAD, the filter paper was dropped so that the LAMP reaction could flow into the detection channel zone without leakage of the flow samples. Five different patterns of dPAD were shown to immobilize the LAMP reaction at different lengths of migratory distance and could be directly measured by BL visualization. To acquire the suitable loading sample volume in the dPAD, the length difference of migratory distance between the negative and positive LAMP reactions at different parasites’ DNA concentration was evaluated. The optimization of each sample volume is shown in Fig. 2b. The samples were loaded at 0.5, 1.5 and 2.5 µL and the distance lengths of each load volume in millimeter scale (mm) were compared. The mean fluorescent distance of each concentration load volume was 1.78 ± 1.30 (mean ± SD) mm for negative control, 3.78 ± 2.11 mm for 10^5^ parasites/mL and 5.11 ± 2.67 mm for 10^7^ parasites/mL, respectively. The fluorescent distances were significantly differences in negative (N) and two positives (10^5^ and 10^7^) (*F* = 25.85, 79.15, 130.85; *P*-value = 0.001, < 0.001, < 0.001, respectively). The differences of the fluorescent migratory distance between positive and negative results at 0.5, 1.5 and 2.5 µL were 1.00, 2.00 and 3.00 mm with RSD values as 0.00 (*n* = 3), 0.00 (*n* = 3) and 0.00 (*n* = 3), respectively for 10^5^ parasites/mL, and 1.67, 3.34 and 5.00 mm with RSD values as 0.35 (*n* = 3), 1.73 (*n* = 3) and 0.20 (*n* = 3), respectively for 10^7^ parasites/mL. According to the difference of the flow results, the load volume at 2.5 µL demonstrated the longest immobilized length of fluorescent migratory distance on the filter paper as the best migratory distance. Of these, 2.5 µL which gained the highest fluorescent length of reaction flow distance were chosen as a standard load volume for all five patterns of the dPAD.

**Figure 3:**
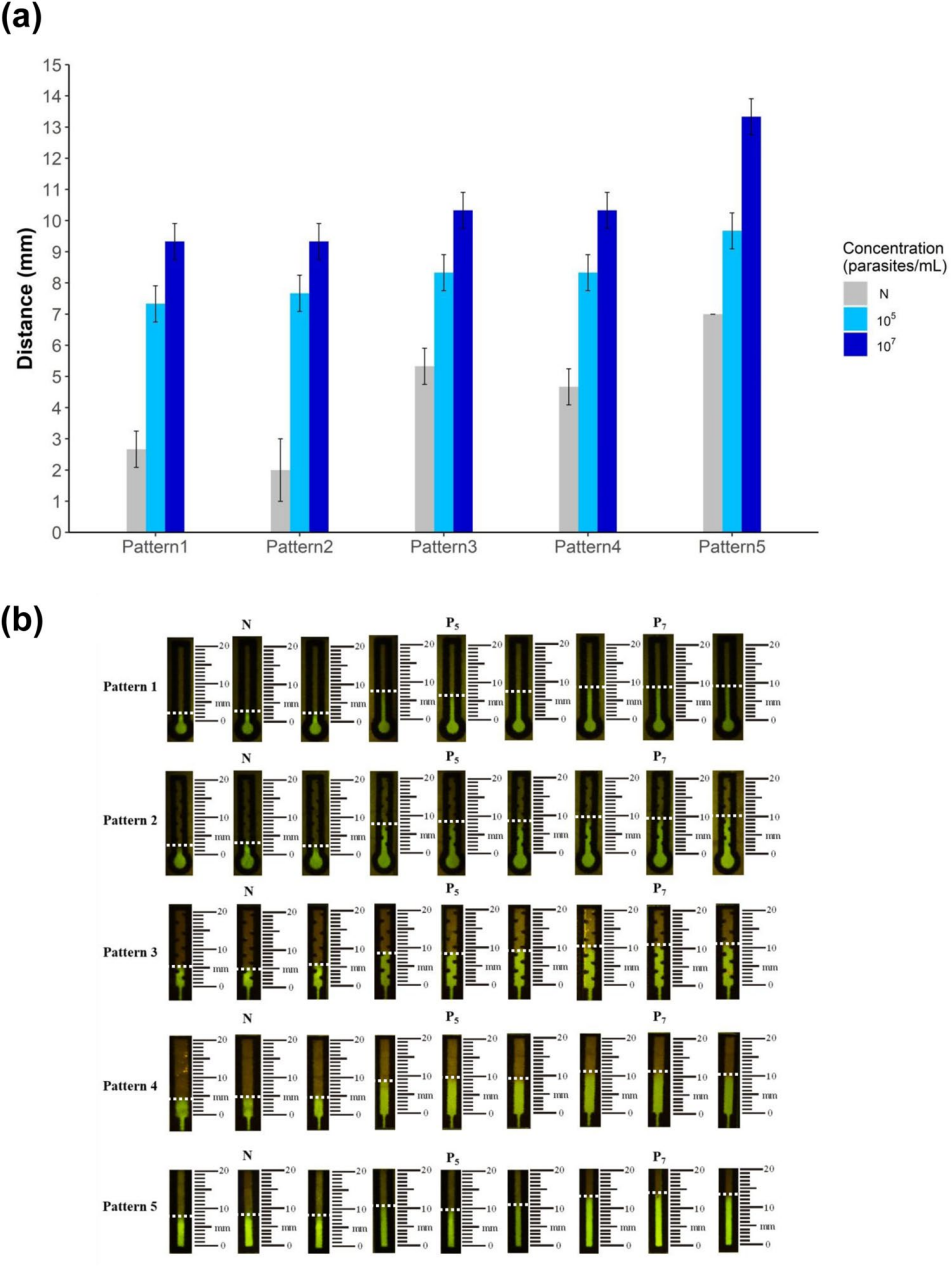
Effect of different patterns of sample and detection channel zones to immobilize migratory distance of LAMP reaction mixture on a distance-based paper device (dPAD), (**a**) mean and standard deviation of fluorescent migratory distance of five different patterns of dPAD, (**b**) fluorescent visualization of migratory distance of five patterns on dPAD. Abbreviations of variable names: no template control (N), positive *Leishmania* template control at 10^5^ and 10^7^ parasites/mL (P_5_, P_7_, respectively).

All five patterns of the designed dPADs could differentiate between negative (N) and positive samples (10^5^ and 10^7^) (Fig. 3). The migratory distances were significantly differences in patterns 1 to 5 (*F* = 7.84, 5.69, 11.44, 9.60, 30.41; *P*-value = 0.026, 0.048, 0.012, 0.017, < 0.001, respectively). The distances of *Leishmania*’ DNA concentration at 10^5^ and 10^7^ parasites/mL were similar to patterns 1 to 4. The differences ranged between 1.67 to 2.00 mm depending on the patterns of the devices (Fig. 3a). However, the highest difference at 3.67 mm was observed in pattern 5 between 10^5^ and 10^7^ parasites/mL (Fig. 3b) that constituted a simple straight pattern in which the LAMP reaction was loaded in the sample channel zone at the bottom of the device and flowed into the detection channel directly straight forward. Thus, pattern 5 was chosen to gain the optimal migratory distance. These were further used to calibrate a device using a linearity between the fluorescent distance and the standard concentrations of *Leishmania*’ DNA, then for quantitative measurement of the LAMP products.

### Semi-quantitative analysis and specificity

**Figure 4:**
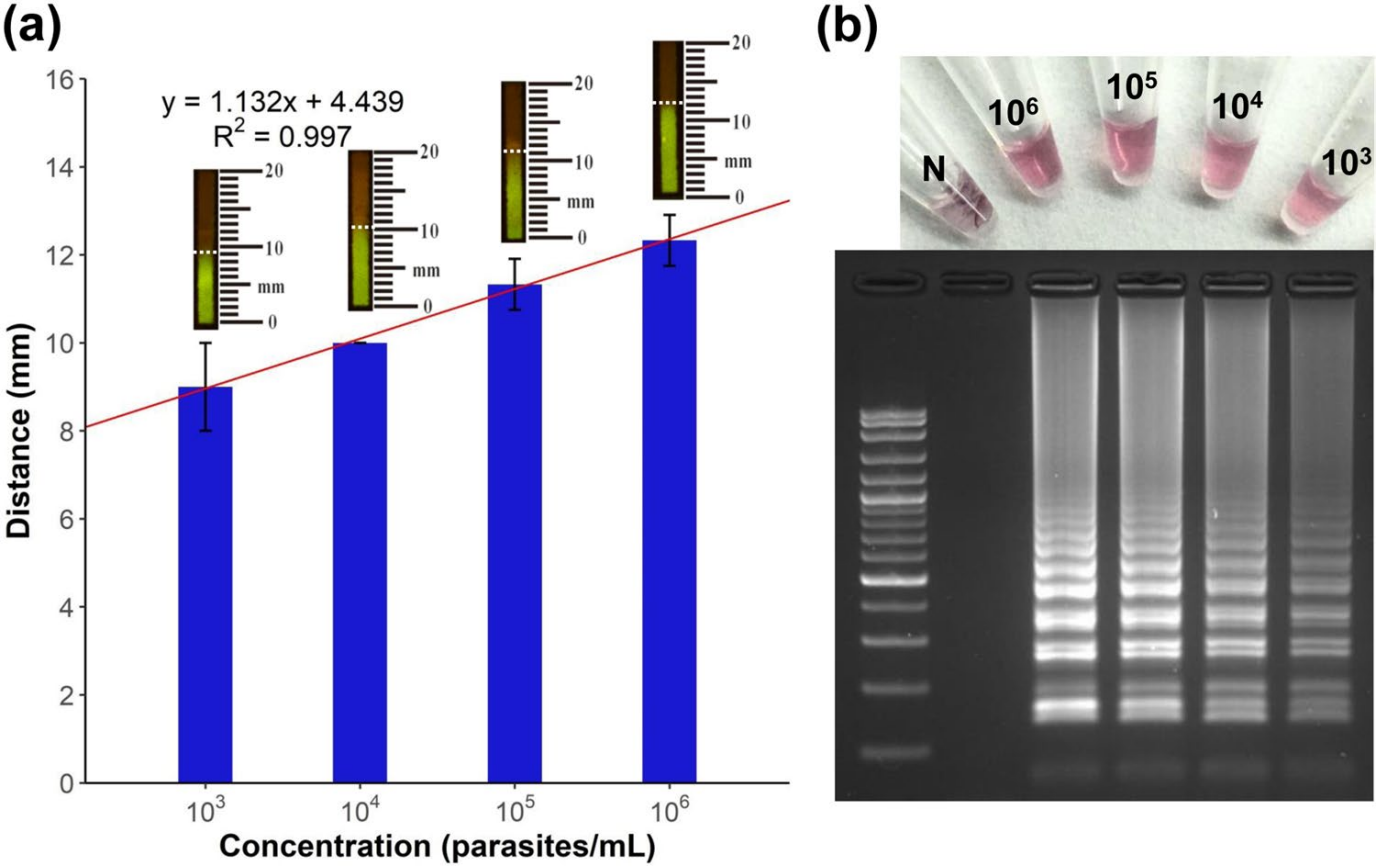
Optimization of semi-quantitative analysis of a distance-based paper device (dPAD), (**a**) semi-quantitative measurement of fluorescent LAMP-dye-complex distance immobilized on dPAD (ten fold serial dilution; 10^6^–10^3^ parasites/mL), (**b**) detection of LAMP products at different concentration of *Leishmania*’ DNA using colorimetric precipitate of specific AuNP-LAMP probes and stem-loop DNA of LAMP amplicons in gel electrophoresis with 100 bp ladder: no template control (N).

Detection of *Leishmania* in the dPAD and specific AuNP-probe, evaluated by tenfold serial dilution from 10^6^ to 10^3^ parasites/mL, were used to observe the calibration curve and linear range (Fig. 4). The results of serial dilution showed concordance of detection between the visual inspection of fluorescent dPAD (Fig. 4a) and colorimetric precipitate AuNPs probe, as well as gel electrophoresis (Fig. 4b). Gel electrophoresis exhibited various multiple bands of stem-loop DNA structures inferring successful amplification of LAMP products. The distance values of fluorescent migratory obtained from the dPAD at each concentration were plotted for observing the calibration curve and linear range. A calibration of the device showed a linearity between the fluorescent migration distance and *Leishmania*’s LAMP amplicons in the range of 10^3^ to 10^6^ parasites/mL with a linear equation as y = 1.132x + 4.439 and R^2^ as 0.997 (Fig. 4a).

**Figure 5:**
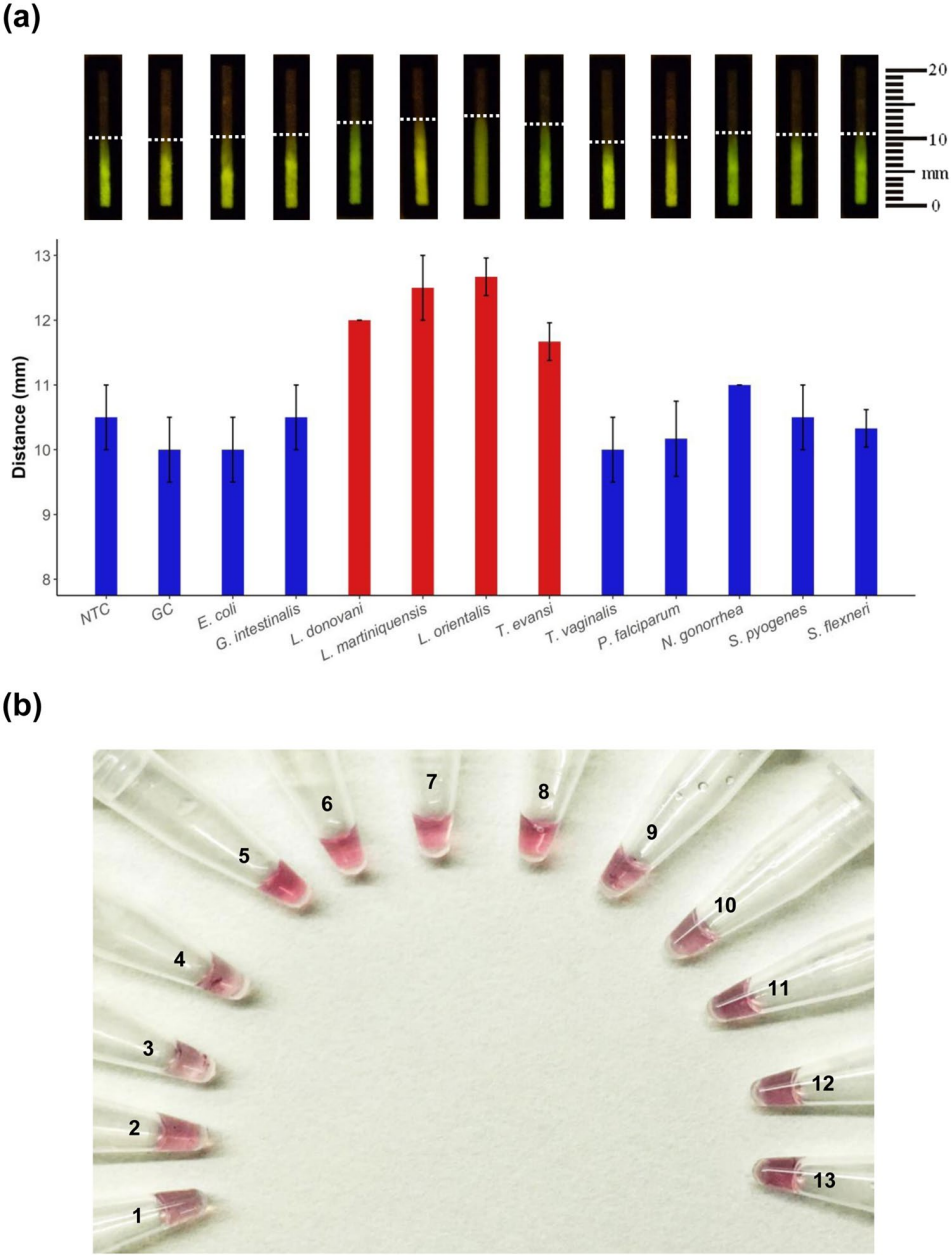
Specificity of semi-quantitative distance-based paper device (dPAD) combined with SYBR safe and AuNP-LAMP assay, (**a**) detection and migratory distance of LAMP amplicons of *Leishmania*’ and different pathogens’ DNA using dPAD, (**b**) LAMP detection of different pathogens’ DNA based on probe-SA-AuNP: tube 1; no template control (NTC), tube 2; human genomic DNA (GC), tubes 3–13; *E. coli*, *G. intestinalis*, *L. donovani*, *L. martiniquensis*, *L. orientalis*, *T. evansi*, *T. vaginalis*, *P. falciparum*, *N. gonorrhea*, *S. pyogenes* and *S. flexneri*, respectively.

Using visual inspection of fluorescent dPAD and colorimetric precipitate AuNPs probe, cross amplifications were not detected except *T. evansi* when non*Leishmania* pathogens’ DNA was tested against the LAMP primers and probe. Obviously, differences in the fluorescent migratory distance were found in the LAMP positive reaction (11.7–12.7 mm) as compared with those of the negative control or reaction mixtures of non*Leishmania* pathogens’ DNA (10.0–11.0 mm) (Fig. 5a) in that the specificity could be distinguished and correlated with the results of specific AuNP-LAMP probes as shown in Fig. 5b. The original gel is presented in Supplementary Figure S1.

### Evaluation of sensitivity and specificity of semi-quantitative SYBR safe and AuNP-LAMP assay using dPAD

**Figure 6:**
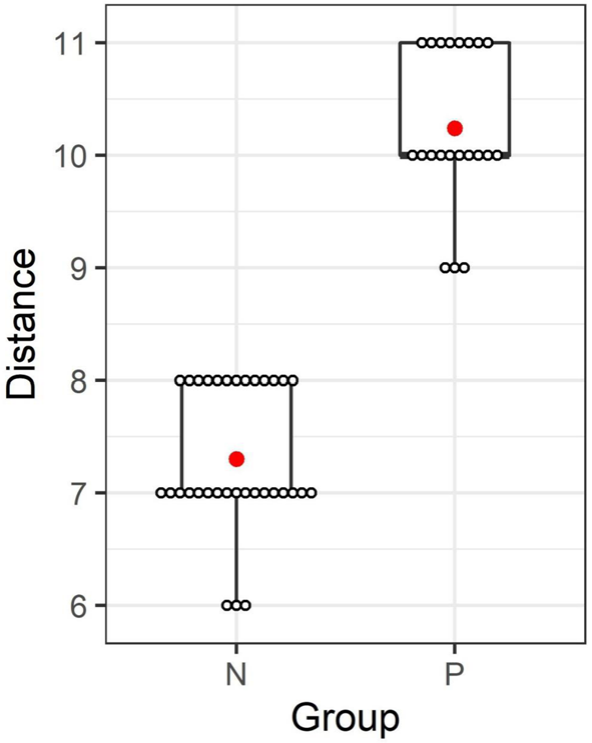
A boxplot representation of length variation of migratory distance (mm) immobilized on a distance-based paper device in an uninfected group (N; *n* = 33) and infected group (P; *n* = 21). Lower and upper boundaries of each box indicate the 25th and the 75th percentile, respectively. Ranges are represented as bars (whiskers) below and above the box, and indicate the 10th and 90th percentiles, respectively. Mean values are marked by the red dot marks on the boxplots. Migratory fluorescent distance length values of each sample (2.5 µL loading volume) were represented by white circle marks (*n*_*uninfected*_ = 33 and *n*_*infected*_ = 21).

Fifty four DNA samples extracted from buffy coat including *Leishmania*/HIV cases (*n* = 22) and uninfected HIV cases (*n* = 32) were used to evaluate and validate the one-step semi-quantitative dPAD combined with SYBR safe screening and specific AuNP-LAMP assay. Using the nested PCR as a reference standard, the sensitivity and specificity of the dPAD were 95.5 (*n* = 21; 95% CI 77.2 to 99.9%) and 100.0% (*n* = 32; 95% CI 89.1 to 100.0%), respectively (Table 1). In addition, the sensitivity of fluorescent detection using SYBR safe screening and colorimetric precipitate detection using posthybridization of AuNP-LAMP probes were the same as the dPAD, 95.5% (*n*_*fluorescent*_ = 21, *n*_*colorimetric*_ = 21; 95% CI, 77.2 to 99.9%, respectively) but the specificity was lower in only the fluorescent closed tube method, 98.5% (*n* = 31; 95% CI 83.8 to 99.9%). The three different methods of this one-step LAMP assay showed significant results that were in almost perfect agreement when compared with reference standard, nested PCR (*P*-value < 0.001, < 0.001, < 0.001; Kappa = 0.92, 0.96, 0.96, respectively). However, the dPAD was in almost perfect agreement when compared with fluorescent closed tube and colorimetric precipitate LAMPs (*P*-value < 0.001, < 0.001; Kappa = 0.96, 0.96, respectively). According to the migratory distance immobilized by the device, the length of the dPAD could be semi-quantified and classified in two levels: the uninfected (7.30 ± 0.64; *n* = 33) and infected groups (10.23 ± 0.70; *n* = 21), respectively (Fig. 6). A significant difference in the fluorescent migratory distance between uninfected and infected groups was observed at a 95% confidence level (uninfected vs infected, *t*-test, *P*-value < 0.001).

**Table 1:** Sensitivity and specificity of prereaction detection using nonspecific SYBR safe indicator and postamplification detection using specific AuNP-LAMP probes and quantitative dPAD in single LAMP assay to detect *Leishmania* DNA among patients with asymptomatic leishmaniasis.

LAMP methods	No. (*n*) of samples with nested PCR^a^		(%) Sensitivity (95% CI)	(%) Specificity (95% CI)	(%) PPV (95% CI)	(%) NPV (95% CI)
	Positive	Negative				
**Prereaction detection**						
Fluorescent closed tube^b^			95.5 (77.2–99.9)	96.9 (83.8–99.9)	95.5 (77.2–99.9)	96.9 (83.8–99.9)
Positive	21	1				
Negative	1	31				
**Postamplification detection**						
Colorimetric precipitate AuNP-LAMP probes^c^			95.5 (77.2–99.9)	100.0 (89.1–100.0)	100.0 (83.9–100.0)	97.0 (84.2–99.9)
Positive	21	0				
Negative	1	32				
**Postamplification detection**						
Distance-based paper device (dPAD)^d^			95.5 (77.2–99.9)	100.0 (89.1–100.0)	100.0 (83.9–100.0)	97.0 (84.2–99.9)
Positive	21	0				
Negative	1	32				

^a^Total number of positive results was based on nested PCR (reference standard method).

^b^SYBR safe initially mixed in the reaction before salt induction of postamplification detection was used as a fluorescent indicator for qualitative screening.

^c^Gold nanoparticle was used as a color and precipitate indicator for specific evaluation.

^d^Measurement of fluorescent migratory distance was used to semi-quantify the LAMP amplicons.

## Discussion

The successful development of the dPAD combined with SYBR safe and gold‑nanoparticle probe LAMP assay was achieved to semi-quantify the amount of *Leishmania*’ DNA among patients with *Leishmania*/HIV co-infection. This highly sensitive LAMP determination could be selectively performed using dual indicators, fluorescent SYBR safe and colorimetric precipitate gold-nanoparticle probe, in the one-step LAMP method. The assay could be simultaneously visualized in prereaction detection using fluorescent SYBR safe for rapid screening, and in postamplification detection using AuNP-LAMP probes for specific colorimetric precipitate induction^15^ and the dPAD for semi-quantification of LAMP amplicons using DNA immobilized cellulose Whatman No. 1^12^. For rapid qualitative screening, fluorescent SYBR safe closed-tube LAMP method was used in prereaction detection, whereas, simultaneously in postamplification detection, the specific verification and quantitative evaluation could be further performed to visualize colorimetric precipitate using AuNP-LAMP probes and to detect the degree of *Leishmania* infection using fluorescent measurement of the migratory distance on the dPAD. Obviously, the remaining SYBR safe dye in the postamplification mixture of the AuNP-LAMP method did not interfere with the hybridizing and aggregating of the AuNPs-probe^15^. Furthermore, the fluorescent emission of the dye could be observed in prereaction mixture and semi-quantitative postamplification mixture using the dPAD. For AuNP-probe hybridization to the target of LAMP amplicons, the polymeric network of cross-linked AuNPs was formed to interpret the unchanged red after salt induction^29,30^ but pale purple with precipitates was induced to the aggregate in the absence of LAMP targets regarding no polymeric network^36–38^.

The best interpretation of semi-quantitative analysis was demonstrated in pattern number five with the load sample volume of 2.5 µL that constituted a simple straight pattern of sample and detection channel zones. The LAMP assay normally generates a high yield mixture of various lengths of stem-loop DNA amplicons in the reaction^12^. Owing to physical adsorption and covalent bonding between the amine-terminated nucleic acid and the polysaccharide^39^, the immobilization of DNA on cellulose such as Whatman No. 1 filter paper is useful for the separation and purification of DNA-binding proteins as well as LAMP products^12^. DNA is a strongly anionic molecule at pH 4.0 or above, and the charge interaction between DNA and the immobilized cationic polymers on cellulose has been reported^40^. The cellulose Whatman No. 1 filter paper is generally available and commercially affordable in all laboratories. Therefore, the filter paper is comparatively suitable and cost effective for fabricating the device to detect and semi-quantify LAMP products^12^. Unlike the qPCR method that can directly measure the amounts of DNA at each amplification cycle, the device indirectly quantify LAMP amplicons after final amplification. Thus, the migratory distance is limited and depends on the final LAMP products at each reaction. The quantitative measurement using migratory distance depended on the final LAMP products at each reaction. When the DNA precursor such as dNTPs was exhausted, the amplification would stop producing loop DNA and enter the plateau phase of LAMP^12^. At this upper limit level, the length of fluorescent migratory distance would be equal and not reflect the true amount of initial parasites’ DNA concentration as well as infection density. Thus, the semi-quantitative measurement could be done by the dPAD to avoid unambiguous naked eye comparison between positive and negative samples using the millimeter scale of the device. The fluorescent distance of the no template control would represent the residual unbinding SYBR safe in the reaction that has comparatively lower anionic. Wax-printed pillars as hydrophobic barriers of microfluidics could delay the flow and increase the sensitivity of the device^41^.

In spite of the unambiguous interpretation, the results of serial dilution showed concordance of the LAMP detection between visual inspection of fluorescence using the device for measuring *Leishmania* density and colorimetric precipitate AuNPs probe as a second clear-cut indicator for LAMP amplicon control^15^, as well as gel electrophoresis. The limitation of sensitivity of the SYBR safe closed tube and colorimetric precipitate AuNP-LAMP probes methods were previously reported to 10^2^ parasites/mL or 0.0147 ng/µL^15^, exhibiting ten times more sensitivity than those of MG and SYBR safe assays^11,14^, and was equal to a simplified closed tube assay, SYBR green I^16^. To gain linearity between the fluorescent migration distance and *Leishmania’*s LAMP amplicons, the range 10^3^ to 10^6^ parasites/mL was used except without template control (N) because the value of the mean migratory distance was underestimated probably due to no LAMP amplicons in the reaction mixture that could demote physical adsorption and covalent bonding on the dPAD. The pan-*leishmania* LAMP primers could detect *L. orientalis*, *L. martiniquensis* and *L. donovani* based on high-copy-number 18S rRNA gene. The pan-*leishmania* LAMP assay is suitable and cost-effective for *Leishmania* screening because, at present, four *Leishmania* species coinfected with HIV patients in Thailand^3^. Similar to related studies, the amplification and cross reactivity with DNA from *Trypanosoma* sp., a closely related blood protozoan, were observed using pan-*leishmania* primers^11,15,16^. According to different clinical presentations exhibited by trypanosomiasis, the cross-amplification would not significantly lead to misdiagnosis^11^. In co-infected endemic areas, additional LAMP primers specific to *Trypanosoma* spp. could be additionally used to selectively identify the co-existence of these two blood protozoa^42^. Multiplex gene detection in the LAMP assay maybe be possible when the fluorescent dye labeling LAMP primers are specifically synthesized^10^. The sensitivity (*n* = 21, 95.5%) and specificity (*n* = 32, 100.0%) of semi-quantitative dPAD using immobilized filter paper were almost equivalent to those of LAMP methods using fluorescent SYBR safe closed tube (*n* = 21 and 31, 94.1 and 97.1%, respectively) and colorimetric precipitates using posthybridization of the AuNP-probe (*n* = 21 and 32, 95.5 and 100.0%, respectively) for detecting and semi-quantifying *Leishmania*’ DNA among patients with *Leishmania*/HIV co-infection when nested PCR was used as the reference standard. In the reaction mixture with a number of LAMP amplicons, the ambiguous evaluation could be interpreted regarding lower fluorescent intensity emissions. Thus, the SYBR safe closed tube is suitable for rapid screening in the prereaction mixture, whereas specific colorimetric precipitate AuNP-LAMP probes and the dPAD could be used as a second clear-cut indicator and semi-quantitative detection of the parasite density, respectively. Furthermore, the kappa statistical test indicated almost perfect agreement between these LAMP detecting methods. Other samples, e.g., saliva, should be examined to further validate feasibility and simplified use of this one-step semi-quantitative LAMP assay.

The rapid diagnostic test kit (RDT) is simpler and faster than the nucleic acid amplification test (NAAT), however, the sensitivity and specificity are lower. Thus, the NAATs are preferred to detect causative agents. Due to the requirement of costly instruments and well-trained lab technicians, NAATs are inconvenient and may not be affordable to local health service centers. Alternatively, basic equipments are required for LAMP assay, whereas an inexperienced worker could simply achieve the assay using step-by-step operating procedures^15^. Unlike RDTs such as malaria and VL strip tests, LAMP reaction required initial DNA for detection; therefore, DNA extraction is inevitable in the LAMP as well as all NAAT techniques. Various simplified methods have been successfully modified and adapted to the assays to overcome the limitations of the LAMP assay in field sites, for example, selective colorimetric and fluorescent dyes^11,14,16,43^, colorimetric precipitate AuNP-probes^13,15^, lyophilized forms^44^, paper-based devices^12^ and dye capsules^45,46^. Recently, the semi-quantitative LAMP assay was successfully developed to detect *E. coli* in urine using inexpensive filter paper fabrication of dPAD^12^. In this present work, we have demonstrated the applied spectrum of the LAMP technique that could be integrated in a one-step LAMP assay to screen, specifically verify and semi-quantify the *Leishmania* parasites among asymptomatic patients with *Leishmania*/HIV co-infection who usually harbor low parasite levels. A selective detection step using DNA-binding fluorescent dye for closed tube and semi-quantitative dPAD, and colorimetric precipitate of specific AuNP-LAMP probe can be interpreted simultaneously, taking approximately 85 to 95 min after adding a DNA sample that is faster than the standard nested PCR method, 8 to 10 h^3^. The cost of the reagents was not much different, approximately US $5 per reaction, based on the cost in Thailand. However, LAMP assay needs a simple heating device or noninstrument nucleic acid amplification (NINA) that is comparatively cheaper than PCR; especially, qPCR^47^. Using a simplified boiling extraction method^11^, the cost per reaction could be reduced about 70% and DNA preparation could be done within 10 min if DNA samples require short term preservation. By introducing Loop primers (LF, loop-forward and LB, loop-backward), the reaction time could be shorten due to rapid synthesis of LAMP products^48^. During post-reaction preparation step, the risk of contamination could be minimized using a simple closed system cabinet. The study supported that LAMP assays could be modified and adapted to broad spectrum of detection ranging from screening with the closed tube, and second clear-cut specific AuNP-probes to the semi-quantitative dPAD in which transilluminator was required to excite fluorescence as a trade-off for unambiguous interpretation. Thus, the LAMP assay is a very promising approach to facilitate early diagnosis and promote suitable treatment that can reduce severity and prevent progression of the disease. It is comparatively simple to proceed selectively with a simultaneous qualitative and semi-quantitative examination in a single assay without the requirement of sophisticated device, however, the assay is not suitable and sensitive for detecting the very low density of *Leishmania* infection that could be achieved by detecting devices (Table 2) such as strip reader^35^ or electrochemical detection^34^.

**Table 2:** The characteristics comparison of different DNA amplification approaches between LAMP assay in this study, lateral flow assay^35^ and electrochemical detection^34^ for detecting *Leishmania* DNA in human and animal, respectively.

Characteristics	DNA amplification approaches		
	Lateral flow assay	Electro-chemical detection	LAMP assay in this study
Isothermal amplification	+	+	+
**Qualitative examination**			
Color interpretation by naked eye	+	−	+
Fluorescent interpretation by naked eye	−	−	+
Electrochemical detection by electronic device	−	+	−
**Quantitative examination**			
Naked eye	−	−	+
Detecting device	+	+	+
Detection limits (parasites/mL)	10	0.8	10^2^
Linear range (parasites/mL)	0.04–20	0.5–500	10^3^–10^6^

## Conclusion

In this study, we developed a simplified and inexpensive dPAD combined with SYBR safe and gold‑nanoparticle probe LAMP assay to detect and semi-quantify *Leishmania* parasites in buffy coat of asymptomatic patients with *Leishmania*/HIV co-infection using colorimetric precipitate and fluorescence, respectively. The two additional steps of AuNPs-probes hybridization and semi-quantitative immobilized dPAD in postamplification preparation of closed tube SYBR safe LAMP assay required 5 and 10 min to clearly interpret the specific results and indirectly measure the number of LAMP amplicons. The sensitivities and specificities of closed tube SYBR safe, colorimetric precipitate AuNP-probes and the dPAD were equivalent, which were as high as 95.5%. The one-step semi-quantitative dPAD combined with SYBR safe screening and specific AuNP-LAMP assay is selective and suitable for simultaneous multipurpose *Leishmania* detection, for example, rapid direct screening in field environments using the closed tube SYBR safe method to reduce contamination prone^14,17^ or, re-evaluation and verification using posthybridization of the AuNPs-probe method to control and assure the first detection^22,25^ as well as semi-quantitative measurement using the dPAD to evaluate the level of infection for appropriate treatment^12^. These applications could empower either asymptomatic leishmaniasis surveillance among patients with HIV or prevention and control in the future. Hence, the assay could be used as an alternative method in addition to traditional PCR detection, especially in areas with limited resources that is reliably fast and practical for field diagnostics to healthcare services in low resource settings that is easily delivered to end-users, especially in low to middle income countries.

## Materials and methods

### Ethics statement

All participants aged > 18 years were informed and enrolled in the study. Written informed consent was received from all participants before collecting samples and analyzing anonymously. All methods were carried out according to relevant guidelines and regulations. This study was approved by the Ethics Committee of the Royal Thai Army Medical Department (IRBRTA 952/2562).

### DNA extraction and PCR amplification for clinical samples and *Leishmania* parasites culture

#### Sample preparation

Eight milliliters of EDTA anti-coagulated blood samples were collected from eligible participants visiting the HIV Clinic at Satun Hospital, Satun Province during a 6-month follow-up period to receive antiretroviral therapy (ART)^15,16^. The whole blood specimen was centrifuged at 900×*g* for 10 min to separate the plasma and buffy coat, and was then kept at −20 °C until further used in DNA extraction^15,16^.

Owing to rapid cell growth and differentiation of *Leishmania* promastigotes in nutritious Schneider’s *Drosophila* medium (Sigma-Aldrich, St. Louis, MO, USA) supplemented with 20% heat inactivated fetal bovine serum (GE Healthcare, Chicago, IL, USA) at 26 °C, a high yield of parasites could be obtained for DNA extraction^15,16^. A total of 10^7^ promastigotes of *L. orientalis* (MON-324; MHOM/TH/2010/ TR), *L. martiniquensis* (MON-229; WHOM/TH/2011/PG) and *L. donovani* (MHOM/ET/67/ HU3) were harvested and washed three times with phosphate buffered saline (PBS) before DNA extraction^15^.

#### DNA extraction

DNA of buffy coat samples was extracted using the Geneaid™ DNA Isolation Kit (blood) (New Taipei, Taiwan), whereas genomic DNA of each species of *Leishmania* parasites was extracted using the DNeasy Extraction Kit (tissue) (Qiagen, Hilden, Germany) according to manufacturer protocols^15,16^. The concentration and quality of the extracted DNA were measured using a Nanodrop spectrophotometer (Denovix, Wilmington, DE, USA) at 260/280 and 260/230 ratios^15,16^. The DNA samples were kept at −20 °C until further use (Fig. S1).

#### PCR amplification and DNA detection

The internal transcribed spacer (ITS1) region of the ribosomal RNA (rRNA) gene of *Leishmania* was amplified based on nested PCR described by Manomat et al.^3^ using a FlexCycler2 Thermocycler (Analytik Jena, Jena, Germany). Promastigotes’ DNA of *L. martiniquensis* (WHOM/TH/2011/PG) was used as a positive control. The PCR products were electrophoresis on 1.5% agarose gel and visualized using a Molecular Imager^®^ Gel Doc™ XR + System with Imager Lab^TM^3.0 (Bio-Rad). Purified PCR products of positive samples were sent to Bionics Co. Ltd. (Seoul, South Korea) for sequencing. The sequencing chromatograms were validated and multiple-aligned with reference *Leishmania* strains retrieved from GenBank using BioEdit, Version 7.0.1.

### Loop-mediated isothermal amplification (LAMP) assay

#### LAMP amplification

The conserved 18S rRNA gene of *Leishmania* was amplified based on LAMP assay as described by Ruang-areerate et al.^15^. A total volume of 25 µL LAMP reaction was prepared. Briefly, each reaction of LAMP mixture consisted of 40 pmol of FIP and BIP primers, 10 pmol of F3 and B3 primers, 1× Thermopol buffer, 8 mM MgSO_4_, 1.4 mM of each dNTP (Biotechrabbit, Hennigsdorf, Germany), 0.8 M betaine (Sigma-Aldrich), 8 U of *Bst* DNA Polymerase Large Fragment (New England Biolabs, Ipswich, MA, USA) and 2 µL of DNA template^15,16^. Additionally, 1× SYBR™ Safe DNA Gel Stain (Thermo Fisher Scientific, Waltham, MA, USA) was added to each reaction mixture^14^ as the nonspecific fluorescent dye indicator of LAMP products. The reaction was incubated at 65 °C for 75 min and then heated at 80 °C for 10 min to inactivate the reaction.

#### LAMP visualization

After incubating, the LAMP amplicons of target DNA were analyzed and confirmed based on direct visual inspection of the reaction tubes using either a blue light (BL) or an ultraviolet light UV transilluminator^14,15^. A positive amplification showed vivid fluorescent emission, whereas no fluorescent emission was observed in the absence of amplification. To determine a mixture of various lengths of the stem-loop DNA of LAMP products, 5 µL of reaction mixture was electrophoresed on 2% agarose gel stained with SYBR™ Safe (Thermo Fisher Scientific) and visualized using an UV transilluminator^15,16^.

### Synthesis of LAMP probe conjugated AuNPs

#### Biotin LAMP probe labeling

The biotin labeling LAMP probes designed by Ruang-areerate et al.^15^ were used to hybridize the *Leishmania* LAMP targets. In brief, the loop forward (LF) region of 18S rRNA LAMP amplicons located between F2 and F1C regions was chosen and designed for specific probes that were complementary to the loop region. Finally, the LF probe was modified by functionalizing with biotin at 5′ region and custom synthesized by Bionics Co. Ltd. (Seoul, South Korea).

#### LAMP probe conjugated AuNPs

As described by Ruang-areerate et al.^15^, a 40 nm particle diameter of colloidal AuNPs stabilized in phosphate buffer that had been conjugated with streptavidin; OD = 10, were commercially synthesized by Kestrel Bioscience Thailand Co. Ltd., Thailand. Previously, streptavidin-AuNPs were diluted at 1:1 with phosphate buffer and subsequently mixed with 100 µM 5′-biotin labeling LF probe at 1:10 ratio by vigorous vortex for 15 to 30 s at room temperature in the dark to obtain 1 nmole streptavidin-AuNP-5′-biotin-probes structure (OD = 0.5). The LAMP probe conjugated AuNPs were then stored in the dark at 4 °C until used^15^.

To detect *Leishmania* LAMP amplicons, the ratio of the hybridization was conducted in a total volume of 15 µL at 65 °C for 5 min^15^. Briefly, the LAMP probe conjugated AuNPs and LAMP reaction mixture were mixed at 1:1 ratio. The concentration of MgSO_4_ was used at 100 mM in a 5 µL fix volume of postamplification mixture (15 µL) to induce aggregation and to visually detect color and colloidal change. A positive result indicated that free AuNP-LAMP probe-amplicon complexes were formed in the reaction remained deep red after incubating, whereas pale purple with dark insoluble precipitate was observed in the absence of LAMP products.

### Device fabrication and quantitative LAMP assay

#### Fabrication of distance-based paper device

The dPADs were fabricated from Whatman filter paper No. 1 (Cole-Parmer, Vernon Hills, IL, USA). Five different patterns of dPADs were designed to semi-quantify LAMP amplicons that were divided in sample and detection zones with channel length 20 mm and width 2 mm using Adobe Illustrator, Version 25.1 (Adobe Inc., San Jose, CA, USA). All designs were printed by laser printer (Xerox ColorQube 8570, Japan) on the filter paper that the hydrophobic wax ink with a width 1 mm that controlled and limited the territory flow of the LAMP reaction sample. Then dPAD was baked at 120 °C for 3 min to induce the wax to penetrate into the filter paper and then cooled at room temperature to limit the boundary of the sample and detection zones of the dPAD. The back side of the device was sealed with a stacked transparent adhesive tape to prevent any leakage of the flow samples^12^.

#### Optimization of sample volume and pattern selection

To optimize the sample volume for the dPAD, loading volume of LAMP reaction was evaluated at 0.5, 1.5, and 2.5 µL. The sample volume that provided the highest fluorescent length of reaction flow distance regarding SYBR Safe indicator was chosen. The fluorescent distance of each loading volume was undertaken in triplicate. The migratory distances of five patterns of dPADs in triplicates were compared to select the dPAD that gained the clearest fluorescent distances at all concentrations. Linear regression was performed to assess the independent association of fluorescent distance and loading volumes at different concentrations (10^5^ and 10^7^ parasites/mL) as well as fluorescent distance and concentrations of five patterns.

#### Measurement of LAMP amplicons

To semi-quantify *Leishmania* parasites, the LAMP reaction mixture was dropped on the sample zone of the dPAD in which the sample flowed into the detection zone and dried within 10 min at room temperature. The device was evaluated by visual inspection under the blue light (BL) so that the migratory distance of fluorescence was measured in millimeter scale.

### Semi-quantitative analysis and specificity

At the mid log phase, 20 µL of *Leishmania* parasites were collected and resuspended with 20 µL of phosphate buffered saline (PBS) containing 0.2% glutaraldehyde (GE, Healthcare, USA). The total parasite densities (parasites/mL) were counted at 400× magnification under light microscope using a Neubauer Chamber. To calibrate semi-quantitative analysis of the dPAD, purified genomic DNA of *L. orientalis* were tenfold serially diluted from 10^6^ to 10^3^ parasites/mL using a Nanodrop spectrophotometer, and were equivalent to 1.147 µg/µL to 0.147 ng/µL, respectively. The experiments were performed in triplicate, and nuclease-free water was used as negative control.

The positive detection using the paper device was confirmed by colorimetric precipitation of specific AuNP-LAMP probes and gel electrophoresis. The optimal volume of LAMP reaction mixture was pipetted to the sample zone of the dPAD as described previously. To achieve a ready use of the readout measurement, the millimeter scale was printed on the right side of the device. Then the mean distance of all LAMP reaction mixtures including negative and tenfold serial dilution of *Leishmania*’ DNA samples were substituted into a linear equation of calibration curve to calculate correlation coefficient (R^2^) of the visual readout obtained from the semi-quantitative fluorescent distance-based paper device.

To ensure that AuNP-LAMP probes were specific to *Leishmania* and cross-amplification with other pathogens was unlikely, the LAMP reactions were examined against human genomic DNA extracted from buffy coat and non*Leishmania*’ DNA including genomic DNA extracted from *Escherichia coli*, *Shigella flexneri*, *Streptococcus pyogenes*, *Neisseria gonorrhea*, *Plasmodium falciparum*, *Trichomonas vaginalis, Trypanosoma evansi* and *Giardia intestinalis* as described by Ruang-areerate et al.^15^. Extracted DNA of three different *Leishmania* species (*L. orientalis*, *L. martiniquensis* and *L. donovani*) were used as positive controls, and nuclease-free water was used for no template control (NTC)^15^.

### Evaluation of sensitivity and specificity of semi-quantitative SYBR safe and AuNP-LAMP assay using dPAD

Sensitivity and specificity of SYBR safe screening, specific AuNP-LAMP probes and semi-quantitative dPAD were determined using a total of 54 genomic DNA samples from buffy coat, consisting of 22 confirmed asymptomatic VL and 32 uninfected cases. Diagnosis of VL was confirmed when nested PCR targeting the ITS1 region of rRNA gene was positive and DNA sequence of the PCR amplicons was identical to *Leishmania*’ DNA. Nested PCR results are considered a reference standard method due to the unavailability of any gold standard^15,16,49^. These data were used to validate and evaluate the sensitivity and specificity of SYBR safe, specific AuNP-LAMP probes and dPAD in one-step LAMP assay. The strength of the agreement was determined between prereaction detection, including fluorescent closed tube (SYBR safe), and postamplification detection using AuNP-LAMP probes and the dPAD. LAMP methods were assessed using the kappa statistical test at 95% confidence intervals (CI) and *P*-value < 0.05 was considered statistically significant. The analysis was performed using STATA, Version SE14 (Stata Corporation, College Station, TX, USA).

## Supplementary Information

Supplementary Figure S1.

## Data Availability

The datasets generated during the current study are available from the corresponding author on reasonable request.
