# Supplementary material for: Distance-based paper device using combined SYBR safe and gold nanoparticle probe LAMP assay to detect Leishmania among patients with HIV
Source: Sci Rep. 2022 Aug 26;12:14558. doi: 10.1038/s41598-022-18765-w (PMC9418321; doi:10.1038/s41598-022-18765-w)

**Supplementary Information**

# Distance-based paper device using combined SYBR safe and gold nanoparticle probe LAMP assay to detect *Leishmania* among patients with HIV

Toon Ruang-areerate[1*], Natkrittaya Saengsawang[2], Panthita Ruang-areerate[3], Nalin Ratnarathorn[4], Thanyapit Thita[1], Saovanee Leelayoova[1], Suradej Siripattanapipong[5], Kiattawee Choowongkomon[6*] and Wijitar Dungchai[2*]

[1]Department of Parasitology, Phramongkutklao College of Medicine, Bangkok, 10400, Thailand. [2]Analytical Chemistry, Department of Chemistry, Faculty of Science, King Mongkut's University of Technology Thonburi, Bangkok, 10140, Thailand. [3]National Omics Center, National Science and Technology Development Agency (NSTDA), Pathum Thani, 12120, Thailand. [4]Research Division, Office of Police Strategy, Royal Thai Police, Bangkok, 10330, Thailand. [5]Department of Microbiology, Faculty of Science, Mahidol University, Bangkok, 10400, Thailand. [6]Department of Biochemistry, Faculty of Science, Kasetsart University, Bangkok, 10900, Thailand.

*Address correspondence to Toon Ruang-areerate, Department of Parasitology, Phramongkutklao College of Medicine, Bangkok, 10400, Thailand; Wijitar Dungchai, Department of Chemistry, Faculty of Science, King Mongkut's University of Technology Thonburi, Bangkok, 10140, Thailand; Kiattawee Choowongkomon, Department of Biochemistry, Faculty of Science, Kasetsart University, Bangkok, 10900, Thailand. E-mails: youangtr@yahoo.com; wijitar.dun@kmutt.ac.th; fsciktc@ku.ac.th

**Figure S1. Original images of the gels shown in Figure 4.**

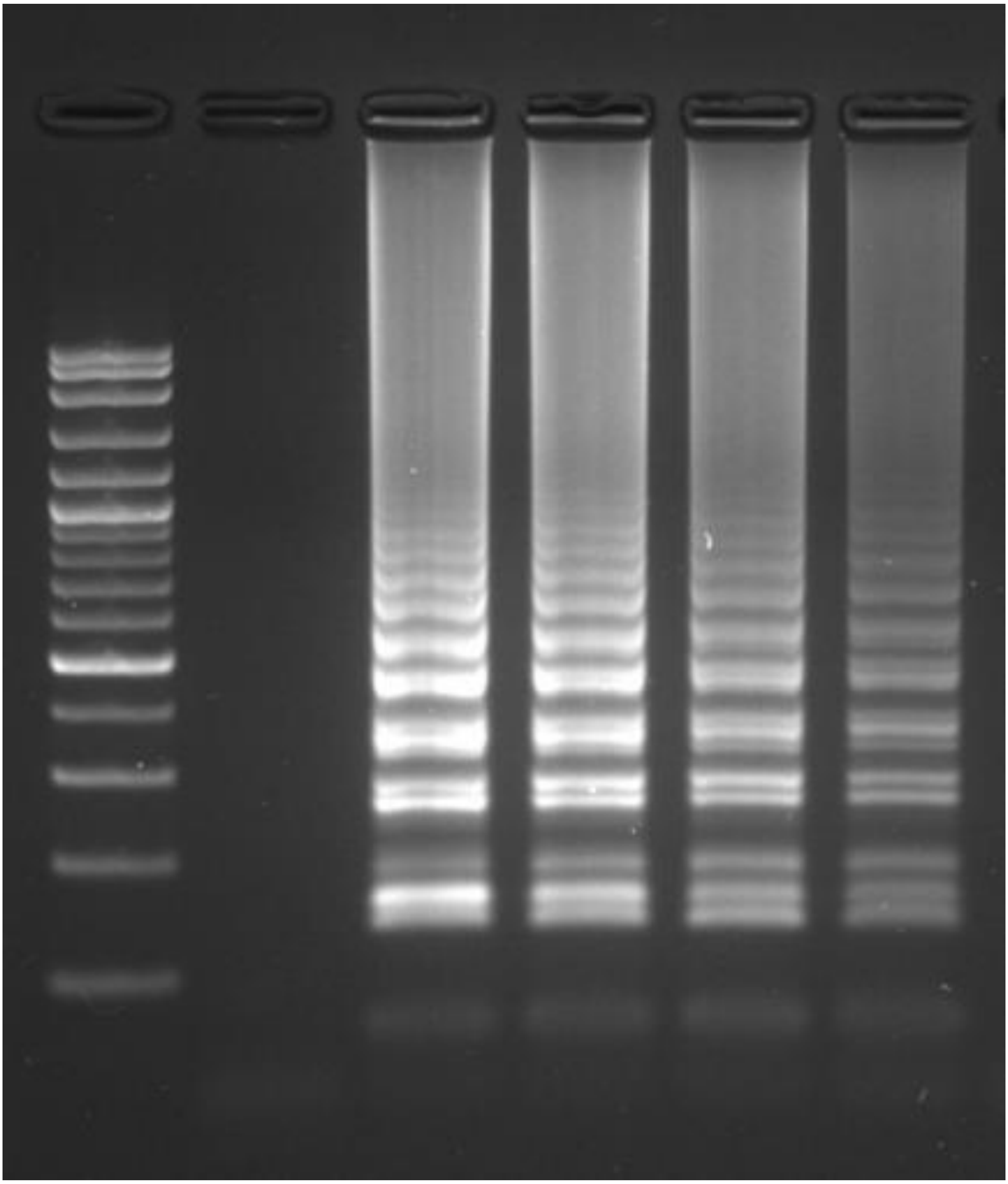

Supplement: Supplementary file 1 — Supplementary Figure S1. [file 41598_2022_18765_MOESM1_ESM.pdf]
